# Astragaloside IV Stimulates Angiogenesis and Increases Nitric Oxide Accumulation via JAK2/STAT3 and ERK1/2 Pathway

**DOI:** 10.3390/molecules181012809

**Published:** 2013-10-16

**Authors:** Shi-Guang Wang, Yan Xu, Jian-Dong Chen, Chuan-Hua Yang, Xiao-Hu Chen

**Affiliations:** 1 First Clinical Medical College, Nanjing University of Chinese Medicine, Nanjing 210023, China; 2 Department of Cardiology, Jiangsu Province Hospital of Traditional Chinese Medicine, The Affiliated Hospital of Nanjing University of Chinese Medicine, Nanjing 210029, China; 3 College of Basic Medical Science, Nanjing University of Chinese Medicine, Nanjing 210023, China; 4 Department of Cardiology, Affiliated Hospital of Shandong University of Traditional Chinese Medicine, Jinan 250011, China

**Keywords:** astragaloside IV, angiogenesis, nitric oxide, JAK2/STAT3, ERK1/2

## Abstract

Astragaloside IV (AS-IV), one of the major active constituents of *Astragalus membranaceus* in Traditional Chinese Medicine, has been widely used to treat ischemic diseases. However, the potential mechanism is this action is unclear. In this study, we tested the hypothesis that AS-IV might promote angiogenesis through multiple signaling pathways. Our data indicate that AS-IV treatment promotes umbilical vein endothelial cells (HUVEC) proliferation, migration, and tube formation. AS-IV treatment also activates JAK2/STAT3 and ERK1/2 signaling pathways, and up-regulates endothelial nitric oxide synthase (eNOS) expression and nitric oxide (NO) production. AS-IV-induced angiogenesis in HUVECs is significantly blocked by specific kinase inhibitors. Our study indicated that AS-IV is a key regulator of NO and angiogenesis through the JAK2/STAT3 and ERK1/2 pathways, which provides a mechanistic basis for the potential use of this compound in the treatment of clinical ischemic diseases.

## 1. Introduction

Angiogenesis, the growth of new blood vessels, has been well known as one of the essential pathological events during diverse chronic ischemic diseases, such as ischemic heart and peripheral disease [1]. Accumulating evidence from both animal studies and clinical trials indicate it could be one of most important intervention strategies to improve prognosis of ischemic diseases by remodeling angiogenesis around the ischemic area [2,3]. Hence, during the last decades, a lot of studies have presented multiple ways to enhance endovascular remodeling and some of them have been translated into clinical patient treatments [4,5].

**Figure 1:**
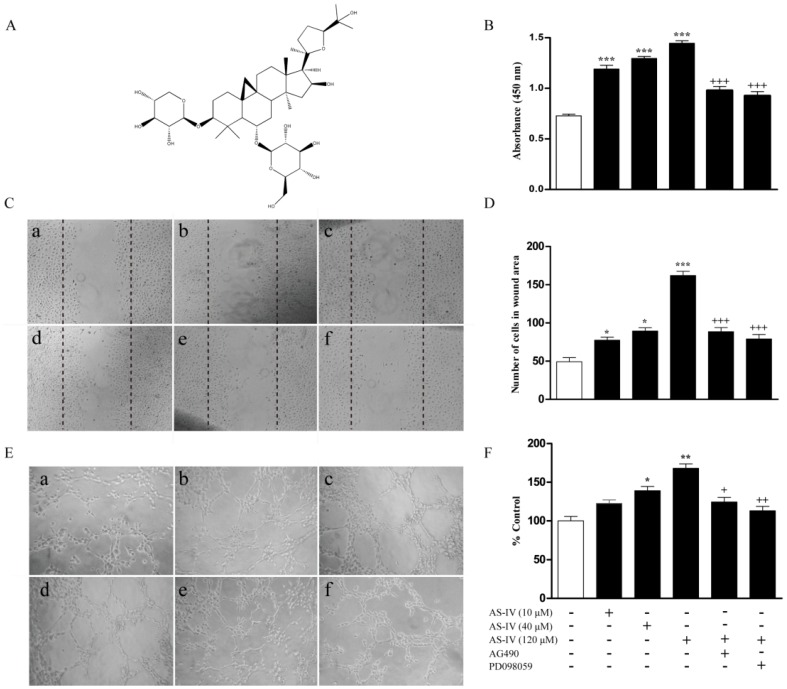
AS-IV stimulates proliferation, migration, and tube formation in HUVECs. (**A**) Chemical structure of AS-IV. (**B**) Serum-starved HUVECs were treated with AS-IV (10–120 μM) for 24 h or pretreated with AG490 (100 μM) and PD098059 (10 μM) for 1 h before incubation with AS-IV (120 μM) for 24 h. Cell growth was assessed for proliferation assay by Cell Counting kit 8 (CCK8). (**C**) Increased migration of HUVECs treated with AS-IV. HUVECs were plated on 6-well plates and then a scratch wound was formed in confluent monolayers. The wound areas were captured after HUVECs were treated with AS-IV (10-120 μM) for 18 h or pretreated with AG490 (100 μM) and PD098059 (10 μM) for 1 h before incubation with AS-IV (120 μM) for 18 h. Shown are representative images (magnification, ×50). (**D**) Quantitative analysis presented as the mean number of cells per wound area. (**E**) AS-IV promoted Matrigel angiogenesis. HUVECs were plated on Matrigel-coated, 24-well plates and treated with with AS-IV (10–120 μM) for 18 h or pretreated with AG490 (100 μM) and PD098059 (10 μM) for 1 hour before incubation with AS-IV (120 μM) for 18 h. Photomicrographs represent the Matrigel tube formation detected by phase-contrast microscopy (magnification, ×200). (**F**) Bar graph shows that AS-IV promoted endothelial tube formation.

Astragaloside IV (AS-IV, 3-*O*-β-d-xylopyranosyl-6-*O*-β-d-glucopyranosylcycloastragenol, Figure 1A), is a saponin that was purified from *Astragalus membranaceus*, which has been widely used for a long time in Traditional Chinese Medicine to treat ischemic diseases [6,7]. The proposed underlying mechanisms include modulation of energy metabolism and Ca^2+^ homeostasis [8], of the activities of antioxidant enzymes such as glutathione peroxidase (GSH-PX) and superoxide dismutase (SOD) [6,7], and dampening of inflammatory-related signals such as nuclear factor-κB (NF-κB) and intercellular adhesion molecule-1 (ICAM-1) [9,10]. Moreover, there is some evidence indicating AS-IV may also regulate angiogenesis via activation of the vascular endothelial growth factor (VEGF) and hypoxia inducible factor-1a (HIF-1a) *in vitro* [11]. Among all of these mechanisms, it is interesting to notice the AS-IV can up-regulate nitric oxide (NO) production, which has been reported by multiple groups, however, there is no direct evidence to demonstrate the involvement of NO pathway in angiogenesis regulation.

In the present study, we used human umbilical vein endothelial cells (HUVECs) to explore the potential mechanism(s) for the effects of AS-IV on angiogenesis, and found that AS-IV stimulated endothelial nitric oxide synthase (eNOS) phosphorylation and NO accumulation by activating the JAK2/STAT3 and ERK1/2 pathways. Our data suggest that AS-IV could potentially be used for chronic ischemic diseases by promoting angiogenesis via the regulation of NO.

## 2. Results

### 2.1. AS-IV Augments Proliferation, Migration, and Tube Formation in HUVECs

HUVECs were cultured and treated AS-IV at various dosages (10–120 μM). Cell proliferation, migration, and Matrigel tube formation assays were used to evaluate *in vitro* angiogenesis as suggested by references [12]. As shown in Figure 1B, AS-IV treatment stimulated the proliferation of HUVECs with a dose-dependent manner. AS-IV also enhanced endothelial cell migration by using scratch assay (Figure 1C–D). Matrigel assays showed that AS-IV induced tube formation in a dose-dependent manner (Figure 1E–F). Moreover, treatment with the JAK2 inhibitor AG490 and the ERK1/2 inhibitor PD098059 significantly inhibited the proliferation, migration, and tube formation induced by 120 μM AS-IV in HUVECs (Figure 1B–F).

(a) control; (b) AS-IV (10 μM); (c) AS-IV (40 μM); (d) AS-IV (120 μM); (e) AS-IV (120 μM) plus AG490 (100 μM); (f) AS-IV (120 μM) plus PD098059 (10 μM). The data are expressed as the means ± SEM (n = 4). * *p* < 0.05, ** *p* < 0.01, *** *p* < 0.001 *versus* control; ^+^ *p* < 0.05, ^++^ *p* < 0.01, ^+++^ *p* < 0.001 *versus* AS-IV (120 μM) group. All the experiments were repeated at least three times.

### 2.2. AS-IV Activates the JAK2/STAT3 Pathway in HUVECs

**Figure 2:**
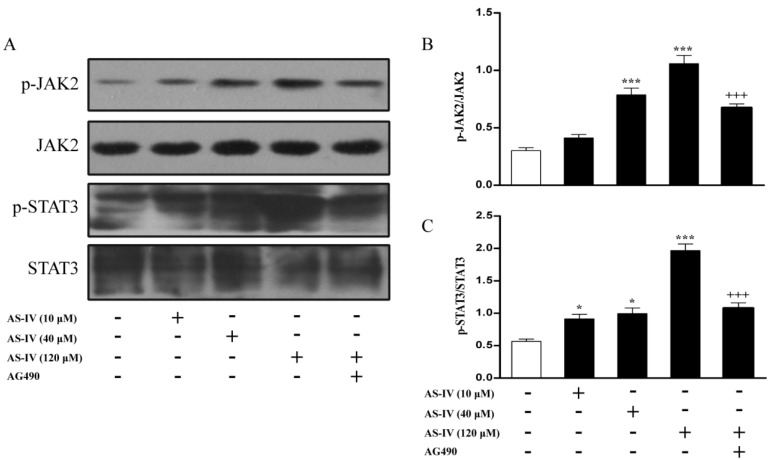
AS-IV activates the JAK2/STAT3 pathway in HUVECs. (**A**) Serum-starved HUVECs were treated with AS-IV (10-120 μM) for 3 h or pretreated with AG490 (100 μM) and PD098059 (10 μM) for 1 hour before incubation with AS-IV (120 μM) for 3 h. Cellular lysate was used to measure phosphorylated JAk2 and STAT3 levels by western blot. (**B**) Bar graph represent p-JAk2/total JAk2 levels. (**C**) Bar graph represent p-STAT3/total STAT3 levels.

Previous studies have shown that JAK2/STAT3 pathway plays an important role in angiogenesis [13,14]. Thus, we investigated the effect of AS-IV on JAK2 and STAT3 phosphorylation using specific phosphorylation antibodies. AS-IV treatment induced a rapid accumulation of phospho-JAK2 and phospho-STAT3 levels (Figure 2A–C), while we did not notice any total protein level changes. Moreover, AS-IV induced kinases activation was significantly blocked by the specific JAK2 inhibitor AG490 (Figure 2A–C). This implicated JAK2/STAT3 pathway might be involved in AS-IV induced angiogenic action in HUVECs.

The data are expressed as the means ± SEM (n = 4). * *p* < 0.05, *** *p* < 0.001 *versus* control; ^+++^ *p* < 0.001 *versus* AS-IV (120 μM) group. All the experiments were repeated at least three times.

### 2.3. AS-IV Promotes the Phosphorylation of ERK1/2, but not JNK and p38 in HUVECs

**Figure 3:**
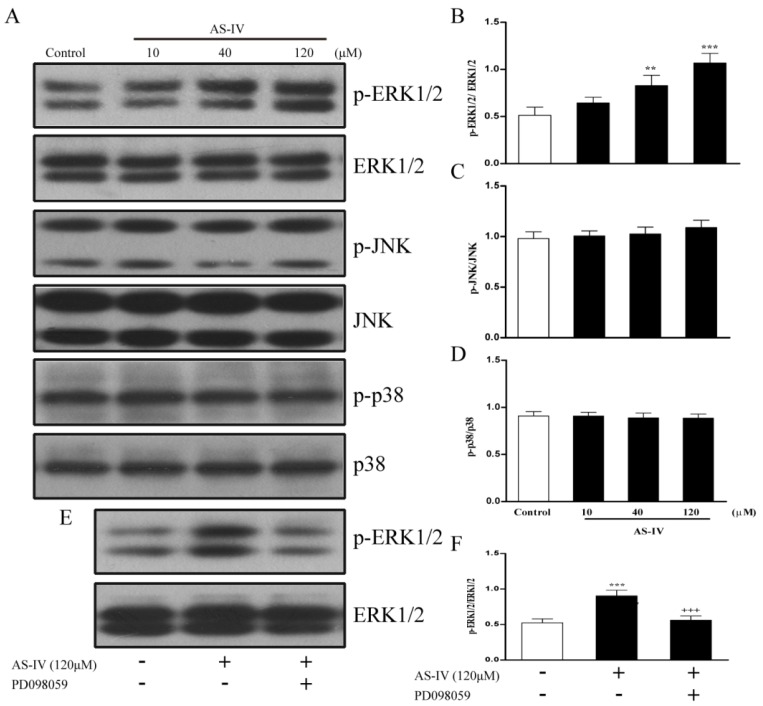
AS-IV promotes the phosphorylation of ERK1/2, but not JNK and p38 in HUVECs. (**A**) Serum-starved HUVECs were treated with AS-IV (10-120 μM) for 1 h. Cellular lysate was used to measure phosphorylated ERK1/2, p38 and JNK levels by western blot. (**B**) Bar graph represent p-ERK1/2/total ERK1/2 levels. (**C**) Bar graph represent p-p38/total p38 levels. (**D**) Bar graph represent p-JNK/total JNK levels. (E) Serum-starved HUVECs were pretreated with AG490 (100 μM) and PD098059 (10 μM) for 1 h before incubation with AS-IV (120 μM) for 1 h. Cellular lysate was used to measure phosphorylated ERK1/2 levels by western blot. (**E**) Bar graph represent p-ERK1/2/total ERK1/2 levels.

MAPK pathways have been documented to be critically involved in endothelial angiogenesis [15]. To clarify the roles of the MAPK signaling pathway in AS-IV induced angiogenic action in HUVECs, we determined ERK1/2, p38, and JNK phosphorylation, and found that AS-IV increased the levels of phosphorylated ERK1/2 in a dose-dependent manner (Figure 3A–D), while phosphorylated p38 and JNK did not significantly change. Furthermore, ERK1/2 signaling was pharmacologically confirmed with the ERK1/2 inhibitor PD098059, which significantly lowered AS-IV induced phospho-ERK1/2 levels (Figure 3E–F).

The data are expressed as the means ± SEM (n = 4). ** *p* < 0.01, *** *p* < 0.001 *versus* control; ^+++^ *p* < 0.001 *versus* AS-IV (120 μM) group. All the experiments were repeated at least three times.

### 2.4. AS-IV Upregulates NO via JAK2/STAT3 and ERK1/2 Pathway

**Figure 4:**
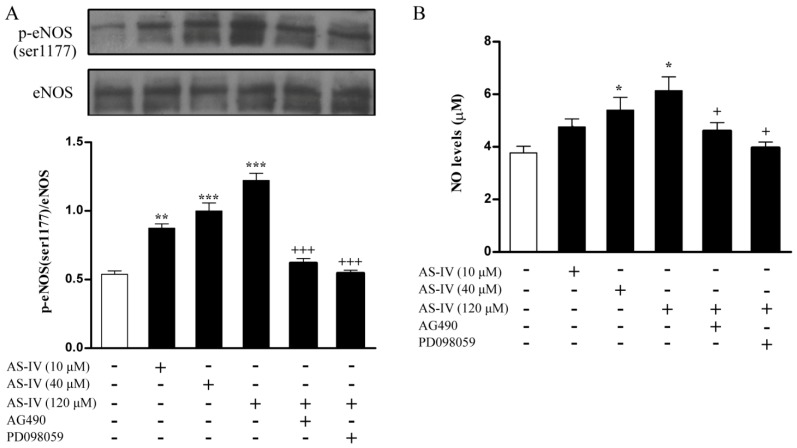
AS-IV upregulates NO via JAK2/STAT3 and ERK1/2 pathway. (**A**) Serum-starved HUVECs were treated with AS-IV (10-120 μM) for 30 min or pretreated with AG490 (100 μM) and PD098059 (10 μM) for 1 hour before incubation with AS-IV (120 μM) for 30 min. Cellular lysate was used to measure phosphorylated eNOS (Ser1177) levels by Western blot. (**B**) Serum-starved HUVECs were treated with AS-IV (10-120 μM) for 30 min or pretreated with AG490 (100 μM) and PD098059 (10 μM) for 1 hour before incubation with AS-IV (120 μM) for 30 min. The stable end product of NO, nitrite, was measured in the medium using the Griess reaction. Medium total nitrite content corrected for protein content of the cell layer was estimated according with the company product.

Lastly, we wanted to explore whether AS-IV induced JAK2/STAT3 and ERK1/2 activation directly regulated NO production and its signaling, which has been indicated by [16,17]. We firstly showed incubation of HUVECs with AS-IV rapidly induced an accumulation of eNOS phosphorylation (Figure 4A). Co-treatment of AG490 or PD098059 significantly decreased AS-IV induced phosphor-eNOS levels (Figure 4A), which indicated activation of eNOS occurred downstream of JAK2/STAT3 and ERK1/2 signaling pathways. Furthermore, co-treatments with either the AG490 or PD098059 significantly decreased AS-IV induced NO levels (Figure 4B).

The data are expressed as the means ± SEM (n = 4). * *p* < 0.05, ** *p* <0.01, *** *p* < 0.001 *versus* control; ^+^ *p* < 0.05, ^+++^ *p* < 0.001 *versus* AS-IV (120 μM) group. All the experiments were repeated at least three times.

## 3. Discussion

In this study we have shown that AS-IV, one of the main active compounds of the traditional Chinese herb *Astragalus membranaceus*, could induce endothelial cell proliferation, migration, and tube formation, which has also been reported in previous studies [18]. We also showed the mechanism of this effect might be through regulating NO via JAK2/STAT3 and ERK1/2 signaling. These data provided a mechanistic basis for the potential application of AS-IV as a candidate angiogenesis modulator during chronic ischemic diseases treatment.

It is well known that NO produced from eNOS plays an important role during physiological and pathological endothelial cells function through regulation of anti-apoptosis, pro-angiogenesis and other mechanisms. Genetic deletion of eNOS in mice impairs angiogenesis function *in vivo* [19,20]. In this study, we found that both eNOS phosphorylation levels and NO production were significantly increased in the AS-IV treated HUVECs comparing with the non-treatment control, which could explain the molecular mechanism of AS-IV-induced *in vitro* angiogenesis. In addition, this experimental system could potentially lead to explore unknown downstream molecules of the NO signaling pathway, by treating endothelial cells with NO donors to increase VEGF [21], or inhibitors of NO synthase such as L-NAME [22]. Moreover, previous studies have demonstrated that AS-IV can increase VEGF mRNA expression in HUVECs [11]. We are also interested in further studies to test if eNOS and NO accumulation are involved in the up-regulation of VEGF in AS-IV-induced angiogenesis.

Moreover, we explored the potential signaling pathway involved in AS-IV-induced eNOS phosphorylation. Specifically, we assessed two major kinase pathways, JAK2/STAT3 and ERK1/2. These two kinase pathways have been shown to regulate eNOS phosphorylation in endothelial cells [16,17]. Incubation of endothelial cells with the JAK2 inhibitor AG490 or the ERK1/2 inhibitor PD098059, abrogated both eNOS phosphorylation and NO production induced by AS-IV. It is interesting to note that activation of JAK2/STAT3 and ERK1/2 are required for angiogenesis, since either JAK2 or ERK1/2 inhibitors reduced AS-IV-induced endothelial cell proliferation, migration, and tube formation. Collectively, our observations highlight the importance of JAK2/STAT3 and ERK1/2 pathways in AS-IV-induced NO production, and both appear to be necessary for HUVEC’s angiogenesis.

## 4. Experimental

### 4.1. Reagents

High purity AS-IV (99% by HPLC analysis) was purchased from National Institute for the Control of Pharmaceutical and Biological Products (NICPB, Beijing, China). The compound was dissolved in dimethyl sulfoxide (DMSO), and the final DMSO concentration did not exceed 0.1% (v/v). Antibodies against ERK1/2 (#9102), Phospho-ERK1/2 (#9101), JNK (#9258), Phospho-JNK (#9251), p38 (#9212), Phospho-p38 (#4511), JAK2 (#3230), Phospho-JAK2 (#8082), STAT3 (#4904), Phospho-STAT3 (#4093), eNOS (#9586) and Phospho-eNOS (#9571) were purchased from Cell Signaling Technology (Beverly, MA, USA). Anti-GAPDH (sc-25778) antibodies were purchased from Santa Cruz Biotechnology (Santa Cruz, CA, USA). AG490, a JAK2 inhibitor, was purchased from Calbiochem (San Diego, CA, USA). PD098059, a MAPK inhibitor, was purchased from Sigma-Aldrich (St. Louis, MO, USA).

### 4.2. Cell Culture

HUVECs were purchased from Shanghai Cell Biology, Chinese Academy of Sciences (Shanghai, China) and were used at passages 2–8 to ensure genetic stability. HUVECs were cultured in DMEM medium supplemented with 10% FBS. The culture was carried out at 37 °C in 5% CO_2_. After the cells reached a confluence of 80%, they were detached using 0.25% trypsin-EDTA. Subsequently, the cells were subcultured once again. The culture media were changed every 2 days.

### 4.3. Cell Proliferation Assay

Cell proliferation was measured using the CCK8 assay (Beyotime Biotechnology, Haimen, China) following the manufacturer’s instructions. In brief, cells were seeded at a density of 2 × 10^4^ per well in 96-well plates and grown overnight. CCK8 reagents were added to a subset of wells under different treatments and incubated for 2 h at 37 °C, after which absorbance was quantified on an automated plate reader (Tecan Group Ltd, Mannedorf, Switzerland).

### 4.4. Cell Migration Scratch Assay

The scratch assay was performed as previously described [23]. Cells (5 × 10^5^) were plated on wells of a 6-well plate and grown to confluency. The cell monolayer was scraped in a straight line with a p200 pipette tip to create a cell-free gap. After washing with serum-free medium twice, cells were treated with AS-IV at 10, 40 and 120 μM for 18 h. In other sets of experiments, cells were pretreated with the JAK2 inhibitor AG490 (100 μM) and the ERK1/2 inhibitor PD098059 (10 μM) for 1 h before incubation with AS-IV (120 μM) for 18 h. Besides, cells receiving DMSO (0.1%) served as vehicle controls, and were equivalent to no treatment. Wound closure was measured by counting the number of cells in the wound area in nine separate replicates in each group. Cells were visualized at × 50 magnification with an phase-contrast microscopy (Nikon, Tokyo, Japan). The images were analyzed using imaging software (NIH Image J, Bethesda, MD, USA).

### 4.5. Tube Formation Assay

The standard matrigel assay was performed as previously described [24]. Cells (8 × 10^4^ cells/well) were seeded on 1:1 (v/v) growth factor reduced Matrigel (BD Bioscience, San Jose, CA, USA) coated 24-well plates, and incubated at 37 °C for 18 h. Quantification of the tubes was performed by taking four 100× images (non-overlapping) of each well, then the closed networks of vessel-like tubes were counted in each image and averaged together. The average of at least four wells was used to determine tube formation for each treatment. Data were analyzed as tube percentage *versus* untreated control wells.

### 4.6. NO Assays

Serum-starved HUVECs were treated with AS-IV (10–120 μM) for 30 min or pretreated with AG490 (100 μM) and PD098059 (10 μM) for 1 h before incubation with AS-IV (120 μM) for 30 min, then conditioned medium was collected and assayed for NO concentrations using QuantiChrom Nitric Oxide Assay Kit (BioAssay Systems, Hayward, CA, USA) according to the manufacturer’s protocol. 

### 4.7. Western Blot Analysis

HUVEC cell lysates were prepared by incubation on ice with lysis buffer (10 mM Tris-HCl, 1 mM EDTA and 250 mM sucrose, pH 7.4, containing 15 μg/mL aprotinin, 5 μg/mL leupeptin, 0.1 mM PMSF, 1 mM NaF, and 1 mM Na_3_VO_4_) and centrifuged at 3,000 × *g* for 15 min at 4 °C. The supernatant was again centrifuged at 12,000 × *g* for 20 min at 4 °C. After resolution of liver protein (equal loading for each sample) by 10% SDS-PAGE, the protein was electrophoretically transferred onto polyvinylidene difluoride membranes (Millipore, Shanghai, China). Membranes were blocked with 5% milk in T-TBS (20mM Tris-HCl [pH 7.5], 135mM NaCl, and 0.1% Tween 20) and then incubated with primary antibodies (1:1,000 dilution) overnight. Membranes were then washed three times with Tris-Tween buffered saline (T-TBS) and incubated with goat anti-rabbit secondary antibody (sc-2004, dilution 1:5,000, Santa Cruz Biotechnology Inc.). Immunoreactive bands were visualized via the enhanced chemiluminescence (Cell Signaling) and quantified via densitometry using imaging software (NIH Image J).

### 4.8. Statistical Analysis

Results were expressed as the mean ± standard error of the mean (SEM). Statistical analysis was performed by a one-way analysis of variance (ANOVA) followed by a Student-Newman-Keul’s. Difference was considered significant at *p* < 0.05.

## 5. Conclusions

In summary, our present study provides mechanistic evidence that AS-IV promotes angiogenesis in HUVECs via NO in a JAK2/STAT3 and ERK1/2-dependent manner. Our present work provides new insight into the angiogenic effect and mechanism of action of AS-IV. However further studies by using *in vivo* and clinical experimental assays are needed to validate these experimental findings.
